# Supplementary material for: The Impact of Telemedicine on Patients with Hidradenitis Suppurativa in the COVID-19 Era
Source: Healthcare (Basel). 2023 May 17;11(10):1453. doi: 10.3390/healthcare11101453 (PMC10218225; doi:10.3390/healthcare11101453)
Supplement: Supplementary file 1 [file healthcare-11-01453-s001.zip › healthcare-2355931-supplementary.pdf]

Supplementary material - the content of the questionnaire used in the study.

1. Select your age: Select only one answer. a. 16 - 20 years old b. 21 - 30 years old c. 31-40 years old d. 41 - 50 years old e. 51 - 60 years old f. 61 - 70 years old g. over 70 years old
2. Select your education level: Select only one answer. a. Elementary school b. Vocational school c. Secondary school d. University degree
3. Please choose your place of residence: Select only one answer. a. Village b. Town up to 50,000 inhabitants c. Town up to 100,000 inhabitants d. Town up to 200,000 inhabitants e. Town with over 200,000 inhabitants
4. Please choose your field of education: Select only one answer. a. Medical b. Non-medical
5. Select your gender: Select only one answer. a. Male b. Female
6. Have you been a patient of the Dr. S. Sakiel Burn Treatment Center in Siemianowice Śląskie? Select only one answer. a. Yes b. No

SURVEY

1. When did you first experience symptoms of the disease? a. Less than a year ago b. Between 1 and 5 years ago c. Between 5 and 10 years ago d. More than 10 years ago (please specify: ....)
2. When was your hidradenitis suppurativa (HS) diagnosed: a. Less than a year ago b. Between 1 and 5 years ago c. Between 5 and 10 years ago d. More than 10 years ago (please specify: ....)
3. Do you think that the disease (HS) was diagnosed too late? a) Yes b) No c) I don't know if it was diagnosed too late
4. How long have you been attending a dermatological clinic? a. Less than a month b. More than a month c. More than six months d. More (please specify: ....)
5. Regarding your wound, on a daily basis: a. I experience pain b. I experience discomfort, but not pain c. I have no pain
6. Please mark on the scale how much your wound hurts on a daily basis:
7. Changing the dressing: a. Is very painful and stressful for me b. Is an uncomfortable situation for me, but not painful c. Doesn't hurt me at all and I don't stress about it
8. Are you able to change your dressings independently or with the help of family/friends? a) Yes, I am able to change dressings independently b) No, I am not able to change dressings independently
9. How do you assess the impact of the SARS-Cov-2 pandemic on access to specialist treatment for your disease, which is hidradenitis suppurativa (HS)? a) I don't see any differences in access to medical services b) There are minor difficulties in accessing specialist treatment c) There are significant difficulties in accessing specialist treatment d) There are very large (huge) difficulties in accessing specialist treatment e) I am unable to see a doctor due to the pandemic
10. Have you experienced any difficulties in accessing specialized clinics recently? a) There were no problems. b) There were significant problems in accessing treatment, such as long waiting times. c) I was unable to access a doctor who provided medical services during the pandemic. d) I treated myself during the pandemic, relying on support groups for HS, such as Facebook forums.
11. Did your health worsen during the pandemic due to the difficult access to specialists? a) My health did not worsen. b) My health slightly worsened. c) My health significantly worsened. d) My health deteriorated very significantly.

12. Have you contracted COVID-19? a) Yes. b) No.
13. Did the COVID-19 infection affect your underlying HS disease? a) Yes, it significantly worsened the condition. b) No, it did not affect the disease at all. c) I do not see a relationship between the deterioration of the disease and the COVID-19 infection.
14. Did you experience any changes in your hospitalization or specialist appointment due to the pandemic? a) No, it never happened. b) Yes, it happened, but it was a marginal phenomenon. c) Yes, changes to appointments were frequent due to the pandemic. d) Yes, my appointment was changed very frequently due to the pandemic. e) I was unable to see a doctor, so I did not receive treatment from specialists during the pandemic.
15. What was the waiting time for a specialist appointment before the outbreak of the pandemic? a) The appointment was scheduled immediately, and the visit took place even on the day of the request. b) > 7 days c) > 14 days d) > 21 days e) > 2 months
16. What is the waiting time for a specialist appointment currently (during the pandemic)? a) The appointment is scheduled immediately, and the visit can take place even on the day of the request. b) > 7 days c) > 14 days d) > 21 days e) > 2 months
17. Have you used teleconsultations? a) No. b) Yes, occasionally. c) Yes, often. d) Yes, very often.
18. How do you rate the quality of services provided through teleconsultations for your HS disease? a) I do not rate it well - my disease requires a thorough holistic approach by the attending physician. b) I rate it very well - teleconsultation is a great way to treat and consult on HS. c) I rate it rather well - teleconsultation is a good idea and helps significantly. d) It is indifferent to me in what form I receive advice.
19. How effective do you consider teleconsultation therapy? a) I rate it very poorly - it is not an effective way to treat HS patients. b) I rate it very well - it is a great way to treat HS patients. c) I rate it rather well - it is a relatively good way to provide services to HS patients. d) It is indifferent to me - the form of advice does not matter to me in my case.
